# PROphylaxis for paTiEnts at risk of COVID-19 infecTion (PROTECT-V)

**DOI:** 10.1186/s13063-023-07128-z

**Published:** 2023-03-13

**Authors:** Toby J. L. Humphrey, Davinder Dosanjh, Thomas F. Hiemstra, Alex Richter, Michael Chen-Xu, Wendi Qian, Vivekanand Jha, Katrina Gatley, Rakshya Adhikari, Francis Dowling, Rona M. Smith

**Affiliations:** 1 Cambridge University Hospitals NHS Foundation Trust, Cambridge, UK; 2 University of Cambridge, Cambridge, UK; 3 Birmingham and West Midlands Lung Research Unit, University Hospitals Birmingham NHS Foundation Trust, Birmingham, UK; 4 Institute of Immunology and Immunotherapy, University of Birmingham, Birmingham, UK; 5 George Institute for Global Health, New Delhi, India; 6 School of Public Health, Imperial College, London, UK; 7 Prasanna School of Public Health, Manipal Academy of Higher Education, Manipal, India

**Keywords:** SARS-CoV-2, COVID-19, Prophylaxis, Niclosamide, Ciclesonide, Sotrovimab, Platform, Trial

## Abstract

**Background:**

Despite the introduction of vaccination, there remains a need for pre-exposure prophylactic agents against SARS-CoV-2. Several patient groups are more vulnerable to SARS-CoV-2 infection by virtue of underlying health conditions, treatments received or suboptimal responses to vaccination.

**Methods:**

PROTECT-V is a platform trial testing pre-exposure prophylactic interventions against SARS-CoV-2 infection in vulnerable patient populations (organ transplant recipients; individuals with oncological/haematological diagnoses, immune deficiency or autoimmune diseases requiring immunosuppression or on dialysis). Multiple agents can be evaluated across multiple vulnerable populations sharing placebo groups, with the option of adding additional treatments at later time points as these become available. The primary endpoint is symptomatic SARS-CoV-2 infection, and each agent will be independently evaluated in real time when the required number of events occurs. Presently, three agents are approved in the platform: intranasal niclosamide, nasal and inhaled ciclesonide and intravenous sotrovimab.

**Discussion:**

Despite the introduction of vaccination, there remains a need for pre-exposure prophylactic agents against SARS-CoV-2. Several patient groups are more vulnerable to COVID-19 disease by virtue of underlying health conditions, treatments received or suboptimal responses to vaccination.

**Trial registration:**

ClinicalTrials.gov NCT04870333. EudraCT 2020-004144-28

## Administrative information

Note: The numbers in curly brackets in this protocol refer to the SPIRIT checklist item numbers. The order of the items has been modified to group similar items (see http://www.equator-network.org/reporting-guidelines/spirit-2013-statement-defining-standard-protocol-items-for-clinical-trials/).

**Table:** 

Title {1}	PROphylaxis for paTiEnts at risk of COVID-19 infecTion (PROTECT-V)
Trial registration {2a and 2b}.	EudraCT Number: 2020-004144-28 Clinicaltrials.gov identifier: NCT04870333
Protocol version {3}	Version 9.0 dated 01 September 2022
Funding {4}	The platform is jointly funded by charitable (LifeArc, Kidney Research UK, Addenbrooke’s Charitable Trust), UK government (NIHR, for the ciclesonide arm) and industry (UNION Therapeutics for the niclosamide arm; GSK/Vir for the sotrovimab arm) sources.
Author details {5a}	1: Cambridge University Hospitals NHS Foundation Trust 2: University of Cambridge 3: Birmingham and West Midlands Lung Research Unit, University Hospitals Birmingham NHS Foundation Trust 4: Institute of Immunology and Immunotherapy, University of Birmingham 5: George Institute for Global Health, New Delhi, India 6. School of public health, Imperial College, London, UK 7. Prasanna School of Public Health, Manipal Academy of Higher Education, Manipal, India
Name and contact information for the trial sponsor {5b}	Mr Stephen Kelleher Cambridge University Hospitals NHS Foundation Trust Email: cuh.research@nhs.net
Role of sponsor {5c}	The trial is sponsored by Cambridge University Hospitals NHS Foundation Trust and University of Cambridge in the UK, and The George Institute for Global Health in India. This is an academic initiated study, and decisions regarding the design and conduct of the study rest with the study team and sponsor. Data will be shared with the relevant funding partners in advance of publication.

## Introduction

### Background and rationale {6a}

Several patient groups are vulnerable to SARS-CoV-2 infection by virtue of demographics, underlying health conditions or as a consequence of treatments for these conditions, and they are at high risk of adverse outcomes. Despite the introduction of widespread vaccination, there remains a need for pre-exposure prophylactic agents. No vaccine is completely effective, new variants of SARS-CoV-2 are emerging and many vulnerable individuals are immunocompromised, either as a result of underlying disease or treatments, and are known to mount a suboptimal response to vaccination against viruses [1–3].

The PROTECT-V trial platform brings greater efficiency, running multiple sub-trials within one master protocol. The core components of the PROTECT-V trial platform include:

- A central coordination trial management team.
- Single-data systems with linkage to UKHSA (formerly PHE).
- Unified sponsor and regulatory oversight.
- Single contract with participating sites for multiple interventions/populations.
- Statistical efficiency—depending on specific interventions/populations, it may be possible to use Bayesian analysis methods that allow adaptive borrowing of information across populations. This will mean in the case that there is a consistent effect across populations, the trial will have greater power to find significant differences for individual patient groups. Furthermore, placebo data may be able to be shared across arms during periods of concurrent recruitment.

At present three agents are approved in the PROTECT-V platform:

1. Intranasal niclosamide
2. Inhaled and intranasal ciclesonide
3. Intravenous sotrovimab

Specific information relevant to each of these agents is provided under sub-headings in each section of this manuscript.

### Objectives {7}

#### Primary objective

The primary aim of the trial is to determine if pre-exposure prophylactic treatment reduces the risk of confirmed, symptomatic SARS-CoV-2 infection in vulnerable immunosuppressed patients.

#### Secondary objectives

The trial also aims to:

1. Determine if pre-exposure prophylactic treatment increases the time to confirmed SARS-CoV-2 infection from the date of randomisation including incidental asymptomatic cases in vulnerable populations
2. Determine the safety of prophylactic treatments in this patient population
3. Determine if prophylactic treatment reduces mortality and severity of COVID-19 disease in the vulnerable populations taking part in the study

### Trial design {8}

PROTECT-V is a platform trial to test pre-exposure prophylactic interventions against SARS-CoV-2 infection in vulnerable patient populations at particularly high risk of COVID-19 and its complications, seeking to identify treatments that either prevent the disease from occurring or reduce the number of cases where the disease becomes serious or life-threatening. In PROTECT-V, multiple agents can be evaluated on the same platform across multiple vulnerable populations, with the option of adding additional treatments at later time points as these become available. The expectation is for as many sites as possible to recruit to all available trial interventions at any time; however, the platform structure and randomisation/data collection systems allow sites to open trial treatment arms according to their capacity.

## Methods: participants, interventions and outcomes

### Study setting {9}

PROTECT-V will be conducted in a hospital setting. Potential participants will be identified via outpatient clinics and dialysis units. The niclosamide and ciclesonide arms will run in approximately 40 UK sites and the sotrovimab arm in 20–30 UK sites. A full list of recruiting study sites will be included in the final study report for each intervention. A parallel niclosamide arm of the PROTECT-V study will also be conducted in India. The George Institute for Global Health, India, will sponsor the PROTECT-V protocol in India. Trial medications and funding for the Indian arm of the study are provided by UNION Therapeutics. The overarching trial electronic case report form (eCRF), statistical support and trial committees will be provided by the UK PROTECT-V trial infrastructure. Data on patients recruited in India will be entered directly into the PROTECT-V study database, and cumulative data from the parallel trial will contribute to the overall sample size and primary endpoint events.

### Eligibility criteria {10}

#### Core eligibility criteria

##### Inclusion criteria

To be included in the trial, the participant must:

1. Be aged 18 years or older
2. Have given written informed consent
3. Be a member of one of the following vulnerable patient populations:
  - *End-stage renal failure on dialysis*—including in-centre haemodialysis, home haemodialysis and peritoneal dialysis
  - *Kidney transplant recipient* receiving at least one of the immunosuppressive medications listed in the protocol
  - *Vasculitis* (according to Chapel Hill Consensus Conference 2012 definitions) or systemic lupus erythematosus (SLE) receiving at least one immunosuppressive medication
  - Glomerulonephritis* receiving at least one immunosuppressive medication

*Glomerulonephritis includes prior histological confirmation of any of the following conditions—minimal change nephropathy, focal segmental glomerulosclerosis (FSGS), IgA nephropathy, primary membranous nephropathy, membranoproliferative glomerulonephritis or lupus nephritis.

##### Exclusion criteria

The presence of any of the following will preclude participant inclusion:

- Inability to provide informed consent or to comply with trial procedures
- COVID-19 at time of enrolment—either positive SARS-CoV-2 swab (PCR or lateral flow test (LFT)) or symptoms highly suggestive of COVID-19 infection
- Known chronic liver disease or hepatic dysfunction as evidenced by ALT or AST > 3× upper limit of the normal range
- Allergy or hypersensitivity to any of the active IMPs or to any of the excipients used
- Pregnant, trying to conceive, unwilling to use contraception or breastfeeding
- Current participation in another interventional prophylactic or vaccine trial* against COVID-19.

*Patients remain eligible for enrolment if they have received SARS-CoV-2 vaccination as part of routine care.

##### Additional exclusion criteria for the niclosamide arm

The following are additional exclusion criteria for the niclosamide arm:

- Significant structural nasal disease in the opinion of the investigator
- Prior participation in the niclosamide arm of the trial (if being re-screened for participation in a second interventional arm)

Additional exclusion criteria for the ciclesonide arm

The following are additional exclusion criteria for the ciclesonide arm:

- Significant structural nasal disease in the opinion of the investigator
- Prior participation in the ciclesonide arm of the trial (if being re-screened for participation in a second interventional arm)
- Currently taking inhaled corticosteroids
- Received a live vaccine within the last 14 days
- Taking one of the following medications: systemic ketoconazole, itraconazole, ritanovir and nelfinavir

##### Additional inclusion criteria for the sotrovimab arm

The following are additional inclusion criteria for the sotrovimab arm:

1. Absent or suboptimal antibody response (defined by Roche Elecsys® Anti-SARS-CoV-2 antibody assay result < 400 AU/mL) to SARS-CoV-2 vaccination or infection
  - Individuals receiving replacement normal human immunoglobulin (NHIg) for any cause of antibody deficiency are eligible regardless of antibody titre, although this will be measured at screening. An Anti-SARS-CoV-2 antibody assay result < 400 AU/mL is NOT required for eligibility in these patients. Antibody titres are unreliable in these patients and vary according to the batch of NHIg administered; timing since NHIg administration and the strict criteria for prescription of NHIg replacement therapy reflects the marked immune compromise of these individuals.
  - Responses should be assessed at least 14 days after the participants’ most recent vaccination.
2. Be a member of an immunocompromised population, which includes but is not limited to those groups listed in the core protocol as well as the following:
  - Immunodeficiency (primary and secondary immunodeficiencies)
  - Any oncology, haematology-oncology or haematology patient who is currently receiving or has received chemotherapy or who is immunocompromised as a result of their disease or treatment
  - Have a diagnosis of an autoimmune/inflammatory disease currently receiving immunosuppression including those individuals currently on prednisolone ≥ 20 mg daily for at least 4 weeks. Those who have received rituximab or alemtuzumab within the last 12 months would also be eligible
  - Solid organ and haematopoietic stem cell transplant recipients

##### Additional exclusion criteria for the sotrovimab arm

The following are additional exclusion criteria for the sotrovimab arm:

In addition to the core exclusion criteria in the master protocol, the presence of any of the following will preclude participant inclusion:

- Antibody response to SARS-CoV-2 vaccination or infection above the pre-specified threshold, as measured by the central laboratory antibody assay (except for individuals on immunoglobulin replacement therapy, who are eligible regardless of antibody titre)
- If in the opinion of the PI it is not in the best interests of the participant to take part in the study—for example, due to limited life expectancy (≤ 12 months) due to pre-existing co-morbidities
- History of hypersensitivity reaction to sotrovimab, one of its excipients or any other monoclonal antibody targeting SARS-CoV-2
- History of receiving any monoclonal antibody targeting SARS-CoV-2 within the last 6 months
- Admission to the hospital for acute, unplanned care at the time of randomisation or in the 2 weeks prior to screening
- History of receiving chimeric antigen receptor T-cell (CAR-T) therapy less than 4 weeks prior to consenting to take part in the study

### Who will take informed consent? {26a}

Consent will be obtained by the principal investigator at each trial site or by a suitably qualified and delegated health professional and member of the research team at each site.

### Additional consent provisions for collection and use of participant data and biological specimens {26b}

Explicit consent for the use of participant data and biological specimens is collected. Participants are asked to agree to the following statement on the consent form “I understand that de-identified information collected during my participation in the PROTECT-V trial may be used to support other, future ethically approved research studies, including research conducted by both commercial and non-commercial organizations in the UK and abroad, and that analysis of the samples may occur that involves DNA/RNA collected from my donated blood samples”. Making information and samples from trials available for further research helps maximise the benefit of conducting trials and allows other researchers to verify the results and avoid duplicating research. Any samples not used will be disposed of in accordance with the Human Tissue Authority codes of practice.

### Interventions

#### Explanation for the choice of comparators {6b}

Each intervention will have a matched placebo as a comparator.

#### Intervention description {11a}

There are currently three separate interventions approved in PROTECT-V: intranasal niclosamide, intranasal and inhaled ciclesonide and intravenous sotrovimab.

##### Niclosamide

Niclosamide, a salicylic acid derivative, is a cheap and safe anti-helminthic medication on the World Health Organization’s List of Essential Medicines that has potent anti-SARS-CoV-2 activity in vitro. It emerged as the leading candidate for activity against SARS-CoV-2 in two separate library screens of existing approved drugs [4]. Furthermore, researchers at Institut Pasteur Korea have reported niclosamide as one of the most potent FDA-approved inhibitors of SARS-Cov-2 in in vitro assays using vero cells, with an IC50 of 0.28 μM > 25× higher than that of chloroquine and > 40× higher than that of remdesivir [5]. Modes of action include modulation of the pH gradient across endosomal membranes inhibiting viral escape, as well as enhanced autophagy through inhibition of S-phase kinase-associated protein 2 [6, 7].

As oral niclosamide is poorly absorbed from the gut with low bioavailability, UNION therapeutics has developed a stable liquid formulation (UNI911). The PROTECT-V trial will administer 1% niclosamide ethanolamine solution via a nasal spray pump twice daily (140 μL of a 1% niclosamide ethanolamine solution, equivalent to 1.4 mg of niclosamide ethanolamine salt per nostril twice daily) and total daily dose of 5.6 mg niclosamide ethanolamine salt (4.7 mg free niclosamide acid) to maximise delivery of drug to the nasal and upper respiratory tract epithelia.

##### Ciclesonide

Ciclesonide is an inhaled corticosteroid licenced for the treatment of asthma and allergic rhinitis that has been shown to possess in vitro anti-SARS-CoV-2 activity but has negligible systemic bioavailability even at higher doses [8]. Class effects of inhaled corticosteroids, particularly at higher doses, include reduced expression of angiotensin-converting enzyme 2 (ACE2) and transmembrane protease serine 2 (TMPRSS2) receptors, which mediate SARS-CoV-2 infection of host respiratory epithelial cells [9]. Inhaled corticosteroids have also been shown to inhibit in vivo production of IL-6, a key pro-inflammatory cytokine in COVID-19 and a major predictor of severe disease and poor outcomes [10–13]. Furthermore, ciclesonide has a specific effect inhibiting in vitro SARS-CoV-2 replication in cultured human bronchial epithelial cells via a novel mechanism on non-structural protein 15 (NSP-15) [14, 15]. These properties make it a plausible candidate for evaluation as a SARS-Co-2 primary prophylaxis intervention in vulnerable patient groups.

The proposed combination of prophylactic inhaled and intranasal ciclesonide will deliver early infection-modifying therapy covering the entire respiratory epithelium, critical in the early stages of COVID-19 [16]. A 320-μg once-daily dose of ciclesonide for the inhaled route has not been shown to be associated with any systemic adverse effects in patients with asthma in the long term, and a dose of 160 μg once daily for the intranasal route is similar to the recommended 200-μg aqueous nasal spray dose, which also has no detectible systemic effects [17–21].

##### Sotrovimab

Sotrovimab is a recombinant human IgG1κ monoclonal antibody (mAb) derived from the parental mAb S309, a potent neutralising mAb directed against the spike protein of SARS-CoV-2 [20]. Sotrovimab contains a two-amino acid Fc modification (termed LS) to increase the half-life and potentially improve bioavailability in the respiratory mucosa through enhanced engagement with the neonatal Fc receptor, which may permit therapeutic concentrations for longer durations [22, 23]. Sotrovimab has also been shown to have potent effector functions in vitro that may result in immune-mediated viral clearance.

The Phase II/III COVID-19 Monoclonal Antibody Efficacy Trial–Intent to Care Early (COMET-ICE) evaluated the efficacy and tolerability of 500 mg sotrovimab administered intravenously in high-risk non-hospitalised patients with mild to moderate COVID-19 infection [24]. In a pre-specified interim analysis including 583 patients, there was a relative risk reduction of 85%; 97.24% confidence interval, 44 to 96; *P* = 0.002 of disease progression leading to hospitalisation or death. The final analysis of 1057 patients showed all-cause hospitalisation or death was significantly reduced with sotrovimab (6/528 [1%]) vs placebo (30/529 [6%]) (adjusted relative risk [RR], 0.21 [95% CI, 0.09 to 0.50]) [25].

Sotrovimab targets a conserved epitope in the SARS-CoV-2 spike protein at a region that does not compete with the binding of the angiotensin-converting enzyme 2. However, pseudotype and live virus neutralisation data suggested reduced neutralisation of Omicron BA.1 and BA.2 confer a 3.5-fold and 35-fold shift in EC_90_, respectively, when compared to the Wuhan reference strain. More recently, sotrovimab was found to neutralise Omicron BA.4, BA.5 and BA.2 to a similar extent [26]. Therefore, a single dose of 2000 mg was selected for the study based on pseudotype and live virus neutralisation data, and sotrovimab clinical PK data from multiple clinical studies evaluating the 500 mg IV dose. Whilst the 2000-mg IV dose is expected to maintain adequate coverage above tissue-adjusted EC_90_ (for wild type and for variants with < 22-fold shift in potency) through 24 weeks, the primary endpoint was changed from 24 to 12 weeks to ensure the highest likelihood of demonstrating efficacy, particularly with the BA.2 variant predominance at the time.

Future prophylactic agents such as alternative monoclonal antibody therapies, antivirals or nasal or inhaled agents may be included within the platform.

#### Criteria for discontinuing or modifying allocated interventions {11b}

Participants will be withdrawn from the trial treatment at the discretion of the PI/CI if continuation in the trial is deemed to be against the participant’s best interest.

Treatment will be withdrawn if:

- Participants become pregnant
- Participant is hospitalised for COVID-19 (patients in the niclosamide and ciclesonide arms will have a final study visit 4–6 weeks after the last dose of IMP; patients in the sotrovimab arm will be followed until week 48)
- Participant experiences unacceptable drug reaction
- Participant has not been followed up for more than 6 consecutive weeks (temporary halt) or for more than 12 consecutive weeks (loss of follow-up and trial withdrawal)

Participants who have been withdrawn from the trial treatment and are experiencing ongoing toxicity will be followed up until the adverse reaction comes to its conclusion. In the event of a participant being withdrawn from the trial treatment, they will continue to receive the most appropriate standard of care treatment available under the guidance of their treating clinician. Following treatment withdrawal, at the final assessment, patients may be re-screened for consideration of enrolment (under a new subject identifier) into one of the other arms of the PROTECT-V trial platform.

Specifically for the niclosamide arm, unacceptable drug reactions which may lead to the withdrawal of treatment include moderate/severe pain or severe burning/itching in the nose, spontaneous nose bleeding as a result of nasal ulceration or oedema which prevents breathing through the nose.

No dose adjustment is required for any of the IMPs (niclosamide, ciclesonide or sotrovimab) even for those individuals with end-stage renal failure on dialysis.

#### Strategies to improve adherence to interventions {11c}

Participants being recruited to the PROTECT-V trial are clinically extremely vulnerable, and therefore, attendance for in-person hospital visits has been minimised where possible. For all IMPs, contact is made remotely with patients every 2 weeks for the duration of the trial. IMP will be collected by the participant during dialysis sessions or sent by courier to the participant to avoid the need for in-person study visits for medication re-supply. For niclosamide and ciclesonide, which need to be self-administered twice daily throughout the study period, participants are asked to complete medication diaries, which are then returned together with IMP containers, for compliance assessment at the final study visit. Specifically for the ciclesonide arm, an easy-to-use delivery method via an AeroChamber spacer and facemask with mouth breathing for the inhaled route and nose breathing for the intranasal route has been employed, which has previously been shown to improve adherence [27, 28].

#### Relevant concomitant care permitted or prohibited during the trial {11d}

All standard-of-care medicines are to continue as per standard practice, including receipt of vaccines against SARS-CoV-2 as part of routine clinical care. Furthermore, should patients contract SARS-CoV-2 infection during the study, they are eligible to receive all standard-of-care treatments both in the hospital and in the community. Any treatment received will be recorded in the eCRFs.

#### Provisions for post-trial care {30}

After the end of the trial, we will endeavour to make available to trial participants further supplies of the IMP in the event that the trial demonstrates benefit, but this cannot be guaranteed.

Cambridge University Hospitals NHS Foundation Trust, as a member of the NHS Clinical Negligence Scheme for Trusts, will accept full financial liability for harm caused to participants in the clinical trial caused through the negligence of its employees and honorary contract holders. There are no specific arrangements for compensation should a participant be harmed through participation in the trial, but no one has acted negligently. The University of Cambridge will arrange insurance for negligent harm caused as a result of protocol design and for non-negligent harm arising through participation in the clinical trial in the UK. The George Institute India will arrange insurance in India.

### Outcomes {12}

#### Primary outcome

The primary outcome for PROTECT-V is confirmed symptomatic SARS-CoV-2 infection during treatment defined as the presence of both:

1. Confirmed SARS-CoV-2 (by either PCR or lateral flow test)
2. One or more symptoms in keeping with COVID-19, including:
  - Respiratory (cough ± sputum and shortness of breath)
  - Constitutional (pyrexia/chills, myalgia/arthralgia, fatigue, rash, headache, confusion)
  - Gastrointestinal (nausea/vomiting, diarrhoea, abdominal pain, loss of appetite)

#### Secondary outcomes

Secondary outcomes include

1. Time to confirmed SARS-CoV-2 infection from randomisation including asymptomatic cases
2. Safety and all-cause mortality
3. Severity of COVID-19 disease (assessed by PI 28 days after the date of positive test)
  - Adapted WHO ordinal scale (defined as the worst category 28 days from the date of positive test or until the date of discharge from hospital, whichever occurred later)
    Healthy carriers—confirmed SARS-CoV-2 infection, no symptoms
    Very mild symptoms, no limitations
    Mild, limitations on activities
    Mild, hospitalised, no oxygen requirement
    Moderate, hospitalised, oxygen via mask or nasal cannula
    Severe, non-invasive ventilation or high-flow oxygen
    Very severe, intubation and mechanical ventilation
    Critical, ventilation and additional organ support (RRT/ECMO)
    Death
  - Length of inpatient stay
  - Common COVID-19 complications (including ARDS, viral pneumonitis, myocarditis/myocardial injury, AKI)

#### Sotrovimab arm

Specifically for the sotrovimab arm, since the IMP is administered as a single infusion on day 1, the primary outcome will be reported at 12 weeks (85 ± 7 days) from the date of IMP administration. The following secondary outcomes will also be assessed: confirmed, symptomatic SARS-CoV-2 infection at 16 weeks (113 ± 7 days), 24 weeks (169 ± 7 days), 36 weeks (253 ±7 days) and 48 weeks (337 ± 7 days) from the date of IMP administration.

### Participant timeline {13}

#### The following is the trial flowchart for the niclosamide and ciclesonide arms:

**Fig. 1:**
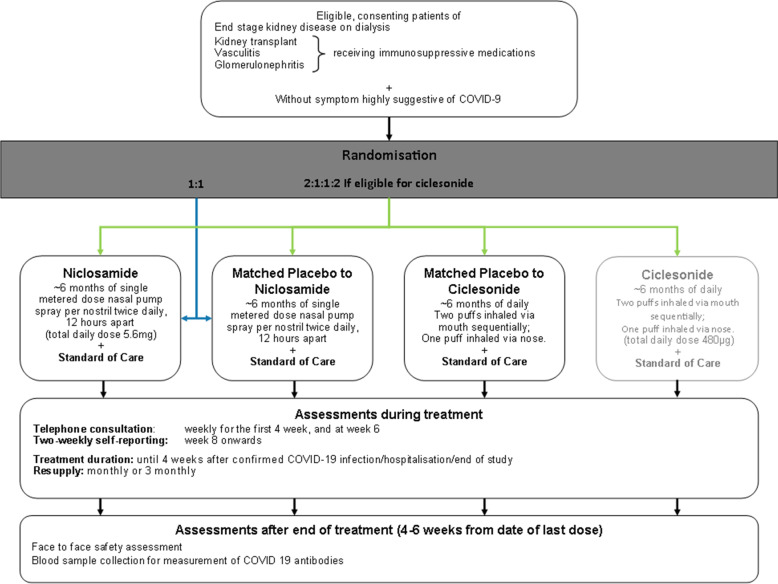
Trial flowchart for the niclosamide and ciclesonide arms

**Fig. 2:**
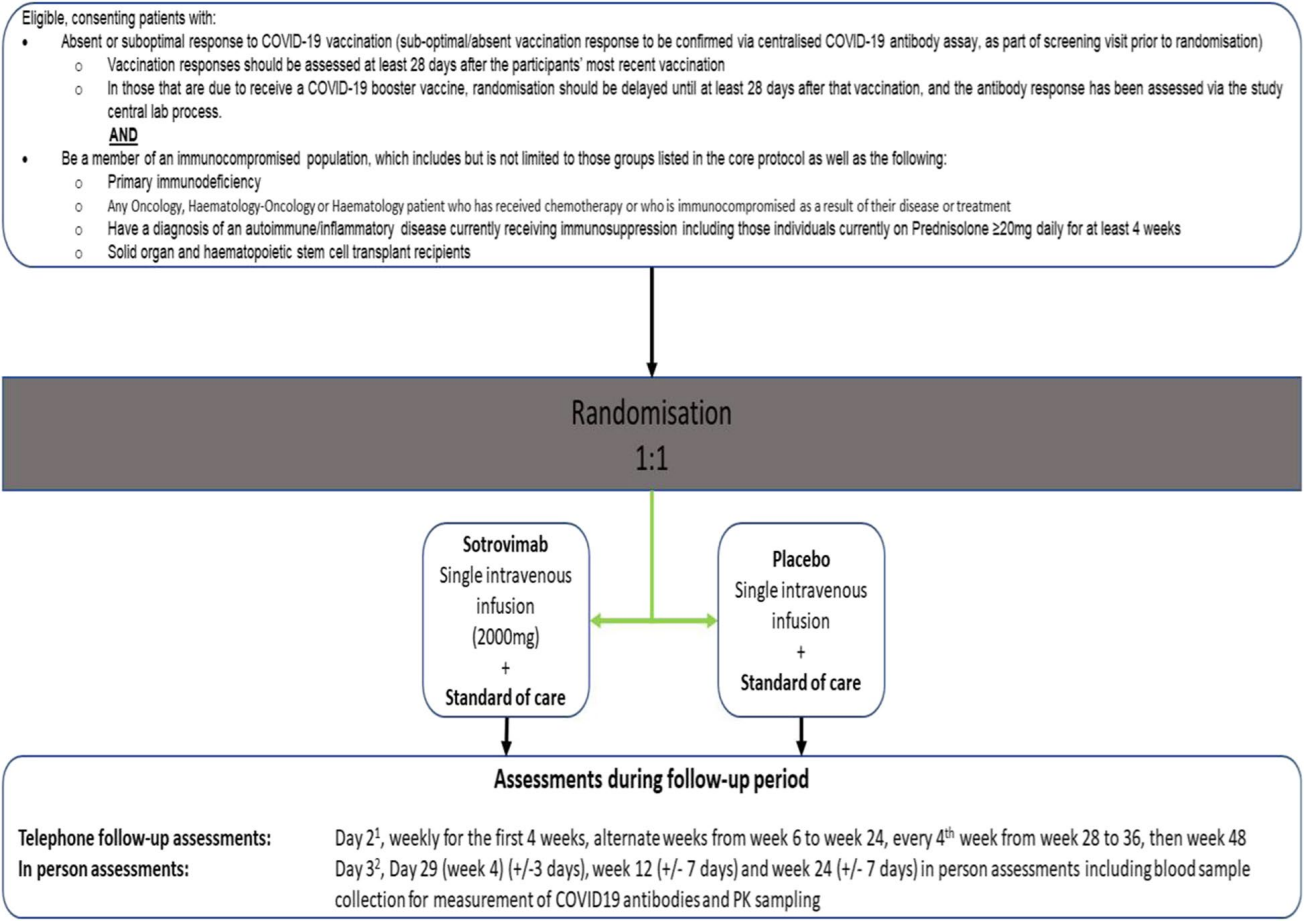
Trial flowchart for the sotrovimab arm

^1^Day 3 (24–72 h post-infusion) telephone call only necessary for lead-in cohorts

^2^Day 4 (48–96 h post-infusion) in-person assessment will only be done for lead-in cohort one and will be a home-visit

The following is the trial flowchart (Figs. 1 and 2) for the sotrovimab arm:

#### Lead in cohorts for the sotrovimab arm

Since there is limited data on a 2000-mg dose of sotrovimab, there will be 3 leads in cohorts.

Infusion and observation times will begin as per cohort 1 (see figure below). After 60 patients have been recruited, a safety analysis will be carried out assessing whether any participants had suffered from a severe infusion-related reaction (IRR) (defined as either requiring IMP treatment termination or hospitalisation for interventional management of an IRR within 24 h).

Any severe IRR in cohort 1 will be reviewed by the independent Data Monitoring Committee (IDMC) to evaluate the IRR and make recommendations to the Trial Management Group (TMG) regarding whether to adjust the infusion time further, implement any other measures or delay progress to cohort 2. If no severe IRRs are noted from the first 60 patients recruited, and the day 4 routine haematological and biochemical assessments do not demonstrate any clinically significant abnormalities, then sites will be instructed to alter the IMP infusion time in line with cohort 2.

After 60 patients have been recruited to cohort 2, data will be reviewed by the IDMC to evaluate the IRR and make recommendations to the TMG regarding whether to adjust the infusion time further, implement any other measures or delay progress to cohort 3. If there is no severe IRR, then sites will be instructed to alter the IMP infusion time in line with cohort 3.

After 60 patients have been recruited to cohort 3, data will be reviewed by the IDMC to evaluate any IRR and make recommendations to the TMG regarding whether to adjust the infusion time further or implement any other measures. If there are no severe IRRs, then the infusion and observation times will remain as per cohort 3 for the remainder of the study.

There will be no pause in recruitment whilst the safety analyses are being carried out between cohorts. Progression to the next cohort will not occur until the safety analysis has been reviewed by the IDMC.

Infusion and observation timings for each lead-in cohort. IMP investigational medicinal product, IRR infusion-related reaction

^1^Day 4 post-infusion, routine haematology and biochemistry results will also be used to inform this decision

^2^Post-infusion observation period to remain at 60 min

### Sample size {14}

#### Niclosamide and ciclesonide arms (designed prior to vaccination roll out)

It is planned to randomise subjects between the prophylactic treatment and placebo or the shared placebo group for patients randomised to more than one prophylactic treatment option. This was based on an estimated 6-month rate of confirmed symptomatic SARS-CoV-2 infection of approximately 15% in the placebo group and 10% in each treatment arm; this would correspond to a hazard ratio of 0.648. With a 0.045 significance level (for an overall significance level of 0.05 with two interim analyses using a Lan-DeMets error-spending approach corresponding to symmetric 2-sided O’Brien-Fleming boundaries and 90% power, the maximum total number of events required would be 235). Allowing for a 15% noncompliance, a total of approximately 1500 subjects would be required.

#### Sotrovimab arm

The primary outcome measure is symptomatic SARS-CoV-2 infection at 12 weeks (85 ± 7 days) from the date of IMP administration. It is anticipated that there will be a reduction of 75% in symptomatic SARS-CoV-2 infection with sotrovimab treatment compared with the placebo arm. With the estimated symptomatic SARS-CoV-2 infection rate at 12 weeks around 2 to 3% in the placebo arm, the table below is a range of sample sizes using the Fisher’s exact test with a 5% significance level, 90% power and two-sided test to detect a 75% reduction. It is planned to recruit a minimum of 1760 participants.

**Table:** 

Symptomatic COVID-19 infection rate at 12 weeks		Total number of patients required	Total sample size allowing for 10% non-compliance
Placebo arm	Sotrovimab arm with a 75% reduction		
2%	0.5%	2380	2650
2.25%	0.5625%	2110	2350
2.5%	0.625%	1900	2120
3%	0.75%	1580	1760

Due to significant uncertainty in the anticipated infection rate, the sample size assumption will be monitored and reviewed regularly by the independent Data Monitoring Committee (IDMC) (details are specified in the IDMC charter) in a blinded manner and sample size re-estimation may be considered under the recommendation of the IDMC.

#### Recruitment {15}

In March 2021, PROTECT-V was awarded UK Urgent Public Health Status as a nationally prioritised COVID-19 platform trial. The study was initially designed for renal patients, and a key eligibility criterion for the niclosamide and ciclesonide arms are that patients are a member of a renal disease patient population (dialysis, transplant, autoimmune disease—including vasculitis, systemic lupus erythematosus or glomerulonephritis). At the time of the study set-up, it was unclear whether sufficient patients could be enrolled in the UK alone, and so a parallel niclosamide arm of the PROTECT-V study was opened in India with the George Institute for Global Health as sponsor for the PROTECT-V protocol in India. The overarching trial eCRF, statistical support and trial committees are provided by the UK PROTECT-V infrastructure and data on patients recruited in India are entered directly into the PROTECT-V study database, with cumulative data from the parallel trial contributing to the overall sample size and primary endpoint events.

For the sotrovimab arm, the trial eligibility criteria were broadened to include any patient who had mounted a sub-optimal vaccine response either due to their underlying condition or treatment for the condition, thus opening the trial to patients including, but not limited to, the following disease areas—haematology, oncology, immunology, rheumatology and transplantation. In order to effectively expand recruitment into these patient groups, national leads have been appointed to drive recruitment across all centres for their allocated patient cohorts.

### Assignment of interventions: allocation

#### Sequence generation {16a}

Randomisation is carried out using a web-based randomisation system (sealed envelope, https://www.sealedenvelope.com/) accessible via password-protected access. Randomisation is stratified by PROTECT-V disease sub-group, age and site using a stratified block randomisation method.

#### Concealment mechanism {16b}

Immediate allocation of treatment will be performed, with documentation of the decision in a blinded confirmatory email. The notification will state what intervention (niclosamide, ciclesonide or sotrovimab) a participant has been randomised to but not whether active or placebo drug has been assigned.

#### Implementation {16c}

Eligible participants will be randomised by local investigators using the web-based randomisation system (Sealed Envelope, https://www.sealedenvelope.com/).

### Assignment of interventions: blinding

#### Who will be blinded {17a}

PROTECT-V has commenced as a double-blind, placebo-controlled study where neither the participant nor clinician is aware of treatment allocation (whether active or placebo). As additional IMPs are added to the platform, the practicalities of blinding treatment and the need/availability of matched placebo will be evaluated on an individual basis. It may be necessary to restrict the number of IMPs in the platform if blinding is to be maintained. The IDMC will regularly review the event rate in individual placebo arms to ensure they are comparable.

#### Procedure for unblinding if needed {17b}

Unblinding must only occur in exceptional circumstances when knowledge of the actual treatment is essential for further management of a participant and/or their safety. If the treating clinician deems unblinding to be necessary, the web-based randomisation system can be used by designated local investigators to unblind. The allocation will be sent by email to the treating clinician and the unblinding notification without allocation should be printed and retained within the investigator site file. An email stating that an unblinding has taken place will be automatically sent to the coordination team and CI for oversight purposes. Unless it is necessary for the safety of the participant, the actual allocation must not be disclosed to the participant or other site personnel, either verbally or by any written correspondence.

### Data collection and management

#### Plans for assessment and collection of outcomes {18a}

Data will be captured using eCRFs and entered directly into a web-based system either by the site team or directly by patients for the symptom checkers in the niclosamide and ciclesonide arms beyond week 6. Sequential eCRF entry is dictated by the data portal to limit errors in the timeline of the trial procedures. For example, eligibility must be confirmed before the randomisation data can be entered, and the follow-up eCRFs are not available until the randomisation data has been entered. Follow-up visits that have specific date windows are checked to be within the specified window. Forms entered into the database are also tracked by the central data manager to flag any outstanding expected forms and ensure that the trial timeline of data collection is adhered to.

Data will also be obtained from the following sources:

- UKHSA (formerly PHE): Data received from the UKHSA will be transferred to the secure data hosting server (SDHS), and SARS-CoV-2 infection data received will be cross-checked with the primary endpoint data provided by the sites.
- SAEs will be reported using the standard trials unit SAE form. Only relevant fields are entered in the trial database
- SARS-CoV-2 antibody samples (for niclosamide and ciclesonide arms—measured at baseline and final trial visit only): results will not be entered directly into the database, but a csv file generated which will be passed directly to the statistician for analysis
- SARS-CoV-2 antibody samples for the sotrovimab arm—samples are processed in real time at the Clinical Immunology Service laboratories in the University of Birmingham Clinical Immunology Service laboratory using the CE marked Roche Elecsys® Anti-SARS-CoV-2 assay (Product code: 09203079190). Screening results are released to site investigators to allow confirmation of eligibility and randomisation. Subsequent measures will be entered into a csv file and passed directly to the statistician for analysis.

### Plans to promote participant retention and complete follow-up {18b}

The study has been designed to minimise patient visits to the hospital during the pandemic. For those participants who withdraw from treatment, but provide consent for ongoing data linkage with UKHSA, data on the occurrence of SARS-CoV-2 infection will be collected (up to 9 months from enrolment for the niclosamide and ciclesonide arms; 48 weeks for the sotrovimab arm) and will be reported enabling an ITT analysis for SARS-CoV-2 infection (although data on symptoms will not be available).

#### Data management {19}

Data will be entered at the site into an eCRF which will be anonymised. All trial data in the eCRF must be extracted from and be consistent with the relevant source documents.

One hundred per cent of variables used to define the primary endpoint and safety data will undergo a cleaning process. For the primary endpoint, symptomatic SARS-CoV-2 infection, the following checks are in place:

1. Confirmation that the trial endpoint form is completed
2. Documentation of a positive SARS-CoV-2 test result by either PCR or lateral flow test. Source data as follows: UKHSA or photographic evidence
3. Cross check primary endpoint form with a symptom checker to ensure symptoms are reported on both
4. Confirmation that the event has occurred whilst receiving or having received IMP

#### Confidentiality {27}

Study participants will provide explicit consent to the use of identifiable data for the purposes of the conduct of the study. Personal identifiable data (PID) will be stored separately from anonymised study data on a secure server hosted within the University of Cambridge School of Clinical Medicine Secure Data Hosting Service. PID will be accessible to the PROTECT-V trial team within the Cambridge Clinical Trials Unit, monitors, auditors and inspectors as required. It is necessary to (1) perform validation of NHS numbers and linkage to routinely collected datasets (UKHSA, NHS Digital, ONS) and (2) generate datasets with participant details for mail merge creation of questionnaires, and is therefore imperative to the conduct of the study.

#### Plans for collection, laboratory evaluation and storage of biological specimens for genetic or molecular analysis in this trial/future use {33}

##### Niclosamide and ciclesonide arms

In view of the pragmatic study design, and aim to minimise study visits, the serum for measurement of antibody titres will only be collected at baseline and the final study visit. The samples will be processed in batch.

##### Sotrovimab arm

Where possible, serum, plasma, PBMC, DNA and RNA samples (up to 70 mL blood in total) will be collected at the day 1 infusion of IMP and in those subjects that develop SARS-CoV-2 infection 28 days later. Additionally, saliva samples will be collected at the Cambridge and Birmingham sites. These samples will be shipped immediately for centrally processing at the University of Cambridge or the University of Birmingham and appropriately stored for future analyses including but not confined to B and T cell immunophenotyping, functional B and T cell assays, anti-cytokine assays, evaluation of the role of mucosal immunity and neutrophil function analyses.

## Statistical methods

### Statistical methods for primary and secondary outcomes {20a}

The following populations will be defined for efficacy and safety analyses for each of the prophylactic interventions.

Intent-to-treat population (ITT) is defined as all participants randomised between the prophylactic intervention and placebo, regardless of whether they actually received trial treatment. The treatment group will be analysed as randomised.

The modified ITT (mITT) population is defined as all participants randomised who received at least one dose of trial treatment. The treatment group will be analysed as randomised at baseline.

The safety population comprises all participants randomised between the prophylactic intervention and the placebo and having received at least one dose of trial treatment. The treatment group will be analysed as treated.

### Efficacy analyses

#### Niclosamide and ciclesonide arms

The primary outcome measure, symptomatic SARS-CoV-2 infection by either PCR or lateral flow test, will be compared between each prophylactic treatment and the randomised placebo groups in the ITT population using a Cox proportional hazards model, considering adjustment for fixed effects with age, sex, ethnicity, patient population and known high-risk pre-existing conditions.

The hazard ratio will be determined and statistical significance will be declared using a 2-sided alpha-level of 0.045 (adjusting for interim analyses). There is no need to use a multiplicity adjustment for the treatment arms as these are considered independent comparisons. A 95% confidence interval for the hazard ratio from the Cox model will be provided. The estimand properties of the primary and sensitivity analyses of the primary endpoint will be detailed in the statistical analysis plan.

For the secondary outcome measure of time-to-confirmed SARS-Cov-2 infection (either by PCR or LFT) in the ITT population, the analysis will use a Cox proportional hazards model as described for the primary outcome measure. The median, 25th and 75th percentile and 95% Cis for time to confirmed SARS-CoV-2 infection (either by PCR or LFT) will be provided. The severity scale of COVID-19 disease will be compared using a proportional odds model for all SARS-CoV-2-infected participants. Length of inpatient stay will be compared using the Fine and Gray approach with discharge alive as an event of interest and hospital death as a competing event for all hospitalised participants. Common COVID-19 complications for all SARS-CoV-2-infected participants in the ITT population will be analysed using the standard chi-square test. The comparisons on secondary outcome measures will be compared according to a pre-specified hierarchal order.

#### Sotrovimab arm

The primary efficacy analysis of this trial will be a logistic regression analysis for the difference in the distribution of symptomatic SARS-CoV-2 infection at 12 weeks between the sotrovimab group and the placebo group (two-sided at an *α*-level of 5%), that is, to test the following hypotheses:

- H0: there is no treatment difference between sotrovimab and placebo in the proportion of patients who develop symptomatic SARS-CoV-2 infection vs. H1: there is a treatment difference between sotrovimab and placebo in the proportion of patients who develop symptomatic SARS-CoV-2 infection.

The odds ratio with the corresponding 95% confidence interval and the relative risk (estimated using the Poisson regression) will be presented. The disease sub-group and age at randomisation will be adjusted for as fixed effects in the analyses.

Primary efficacy analyses will be performed according to ITT in participants who have received any IMP. The analyses will be performed when all patients randomised have been in the trial for 12 weeks or up to 4 weeks post the date of the last confirmed positive SARS-CoV-2 result to allow for symptom collection, for those cases that occur between week 8 and week 12. If the statistical significance at a 2-sided 0.05 level is established for the primary efficacy outcome measure, a hierarchical testing procedure will be applied to the key secondary efficacy outcome measures at a 2-sided 0.05 significance level. The order of testing sequence for key secondary outcome measures is (1) confirmed, symptomatic SARS-CoV-2 infection at 16 weeks (113 ± 7 days) from the date of IMP administration; (2) confirmed, symptomatic SARS-CoV-2 infection at 24 weeks (169 ± 7 days) from the date of IMP administration; (3) confirmed, symptomatic SARS-CoV-2 infection at 36 weeks (253 ± 7 days) from the date of IMP administration; and (4) confirmed, symptomatic COVID-19 infection at 48 weeks (337 ± 7 days) from the date of IMP administration. Further detail will be documented in the statistical analysis plan (SAP).

### Safety analyses

SAEs for all treatment arms will be reported using the preferred term MedDRA system according to treatment group. For the niclosamide and ciclesonide arms, adverse events will be collected via the fortnightly symptom checkers that are completed. Data on infusion reactions, AEs up until day 29 and laboratory parameters, graded according to DAIDS categorisation will be presented for the sotrovimab arm. Also for the sotrovimab arm, data on adverse events of special interest (AESIs) will be collected throughout the study period. These are detailed in the trial protocol.

### Efficacy analyses

#### Niclosamide and ciclesonide arms

The primary outcome measure, symptomatic SARS-CoV-2 infection by either PCR or lateral flow test, will be compared between each prophylactic treatment and the randomised placebo groups in the ITT population using a Cox proportional hazards model, considering adjustment for fixed effects with age, sex, ethnicity, patient population and known high-risk pre-existing conditions.

The hazard ratio will be determined and statistical significance will be declared using a 2-sided alpha-level of 0.045 (adjusting for interim analyses). There is no need to use a multiplicity adjustment for the treatment arms as these are considered independent comparisons. A 95% confidence interval for the hazard ratio from the Cox model will be provided. The estimand properties of the primary and sensitivity analyses of the primary endpoint will be detailed in the statistical analysis plan.

For the secondary outcome measure of time-to-confirmed SARS-Cov-2 infection (either by PCR or LFT) in the ITT population, the analysis will use a Cox proportional hazards model as described for the primary outcome measure. The median, 25th and 75th percentile and 95% Cis for time to confirmed SARS-CoV-2 infection (either by PCR or LFT) will be provided. The severity scale of COVID-19 disease will be compared using a proportional odds model for all SARS-CoV-2-infected participants. Length of inpatient stay will be compared using the Fine and Gray approach with discharge alive as an event of interest and hospital death as a competing event for all hospitalised participants. Common COVID-19 complications for all SARS-CoV-2-infected participants in the ITT population will be analysed using the standard chi-square test. The comparisons on secondary outcome measures will be compared according to a pre-specified hierarchal order.

#### Sotrovimab arm

The primary efficacy analysis of this trial will be a logistic regression analysis for the difference in the distribution of symptomatic SARS-CoV-2 infection at 12 weeks between the sotrovimab group and the placebo group (two-sided at an *α*-level of 5%), that is, to test the following hypotheses:

- H0: there is no treatment difference between sotrovimab and placebo in the proportion of patients who develop symptomatic SARS-CoV-2 infection vs. H1: there is a treatment difference between sotrovimab and placebo in the proportion of patients who develop symptomatic SARS-CoV-2 infection.

The odds ratio with the corresponding 95% confidence interval and the relative risk (estimated using the Poisson regression) will be presented. The disease sub-group and age at randomisation will be adjusted for as fixed effects in the analyses.

Primary efficacy analyses will be performed according to ITT in participants who have received any IMP. The analyses will be performed when all patients randomised have been in the trial for 12 weeks or up to 4 weeks post the date of the last confirmed positive SARS-CoV-2 result to allow for symptom collection, for those cases that occur between week 8 and week 12. If the statistical significance at a 2-sided 0.05 level is established for the primary efficacy outcome measure, a hierarchical testing procedure will be applied to the key secondary efficacy outcome measures at a 2-sided 0.05 significance level. The order of testing sequence for key secondary outcome measures is (1) confirmed, symptomatic SARS-CoV-2 infection at 16 weeks (113 ± 7 days) from the date of IMP administration; (2) confirmed, symptomatic SARS-CoV-2 infection at 24 weeks (169 ± 7 days) from the date of IMP administration; (3) confirmed, symptomatic SARS-CoV-2 infection at 36 weeks (253 ± 7 days) from the date of IMP administration; and (4) confirmed, symptomatic COVID-19 infection at 48 weeks (337 ± 7 days) from the date of IMP administration. Further detail will be documented in the statistical analysis plan (SAP).

### Safety analyses

SAEs for all treatment arms will be reported using the preferred term MedDRA system according to treatment group. For the niclosamide and ciclesonide arms, adverse events will be collected via the fortnightly symptom checkers that are completed. Data on infusion reactions, AEs up until day 29 and laboratory parameters, graded according to DAIDS categorisation will be presented for the sotrovimab arm. Also for the sotrovimab arm, data on adverse events of special interest (AESIs) will be collected throughout the study period. These are detailed in the trial protocol.

### Interim analyses {21b}

#### Niclosamide and ciclesonide arms

The study will be reviewed every 2 months by the IDMC for safety, combined primary outcome measure event rate and making a recommendation for performing efficacy analyses. Should there be sufficient evidence of a difference in the primary outcome measure between the niclosamide arm and the matched placebo arm at one planned interim analysis, the IDMC may consider recommending early termination of the study. As a guide to the IDMC, considering the total duration of the study is around 1 year, a maximum of two formal interim analyses, based on the number of primary endpoint events, are to be performed using a Lan-DeMets error-spending approach corresponding to symmetric 2-sided O’Brien-Fleming boundaries. The study will only be stopped earlier if there is sufficient evidence of benefit using the O’Brien-Fleming boundaries, that is, the value of the test statistic crosses the O’Brien-Fleming boundary of the beneficial effect of niclosamide. As a potential pivotal study, it is not planned to stop the study for futility. The study will be stopped early if there are any safety concerns based on the recommendation of the IDMC and the approval from the Trial Steering Committee (TSC). As guidance, the study might be stopped early for safety concerns

- If the rate of unacceptable toxicity in the niclosamide or ciclesonide arm is over 20%, that is, the estimated lower limit of a 95% confidence interval is greater than 20%. In general, local intolerance should only be regarded as an unacceptable toxicity if the event fulfils the criteria for a serious adverse event or is severe.
- If the incidence of moderate/severe adverse events/symptoms is 15% more in the niclosamide or ciclesonide arm with a minimum of 200 participants in each arm at a significance level of 0.05.

#### Sotrovimab arm

An independent Data Monitoring Committee (IDMC) will review the data, by treatment group on the safety of patients in the trial. The IDMC will meet approximately every 2 months until the end of the trial. In addition, during the initial three lead-in cohort period and prior to the final infusion time being established, the IDMC will review severe infusion-related reaction (IRR) events whenever they are observed and have additional meetings as required.

There are no plans to stop the trial early for efficacy, and therefore, no interim analyses for efficacy or futility. Efficacy data, by treatment arm, will therefore not be routinely provided. The IDMC can request efficacy data, by treatment group, confidentially from the Trial Steering Committee (TSC) if changes to the protocol are considered, and these data are required by the IDMC in order to reach decisions about the ongoing risk/benefit to patients in the trial. If efficacy data are reviewed, a Haybittle-Peto boundary may be used. Full details will be given in the IDMC charter.

### Methods for additional analyses (e.g. subgroup analyses) {20b}

Details will be documented in the statistical analysis plan.

#### Methods in analysis to handle protocol non-adherence and any statistical methods to handle missing data {20c}

Details will be documented in the statistical analysis plan.

#### Plans to give access to the full protocol, participant-level data and statistical code {31c}

The full protocol is available on the study website and is submitted as supplementary material with this manuscript. Participant-level data and statistical code will be shared with the relevant industry partner.

### Oversight and monitoring

#### Composition of the coordinating centre and trial steering committee {5d}

##### Trial Management Group (TMG)

The TMG will meet at least weekly during the initial set-up, and then at least two weekly face to face or by teleconference to oversee the running of the trial thereafter. TMG members will review SAEs which have occurred in the trial. If there are specific safety concerns, these may be raised with the TSC and IDMC. TMG members will include co-investigators, the trial statistician, trial pharmacist, trial coordinator(s) and data manager(s) at the Cambridge Clinical Trials Unit (CCTU).

##### Trial Steering Committee (TSC)

The TSC is responsible for the review of the trial and related activities at regular intervals. The TSC also provides overall supervision for the trial, to ensure that it is conducted in accordance with the protocol and GCP and to provide advice through its independent chairman. The committee will aim to convene at regular intervals to review the data and discuss the recommendations from the IDMC. The details of the TSC are set out in the PROTECT-V Trial Steering Committee Charter.

#### Composition of the data monitoring committee, its role and reporting structure {21a}

The Independent Data Monitoring Committee (IDMC) comprises an unblinded independent group, as defined in the PROTECT-V Data Monitoring Committee Charter document, which defines the role of the IDMC. The IDMC is responsible for the review of all safety data and meets regularly whilst the trial is ongoing, from opening to recruitment until the final visit of the last participant.

#### Adverse event reporting and harms {22}

All adverse events (AEs) are recorded from the point of informed consent regardless of whether a participant has yet received a medicinal product. Individual adverse events are evaluated by the local site investigator for the evaluation of their seriousness and any relationship between the investigational medicinal product(s) and/or concomitant therapy and the adverse event (causality). All expected adverse reactions (ARs) are listed in the latest MHRA-approved version of the specified reference safety information (RSI), and this must be used when making a determination as to the expectedness of the adverse reaction. If the adverse reaction meets the criteria for seriousness, this must be reported to the sponsor within 24 h of the investigator becoming aware of the event.

##### Niclosamide and ciclesonide arms

All AEs and ARs, which are new or significantly worse since commencing the trial, should be recorded on the symptom checker questionnaire in the eCRF and sent to the trial coordination centre within 1 month of the investigator becoming aware of the event.

##### Sotrovimab arm

All AEs (serious and non-serious) are reported until day 29 of the study as these are most likely to be potentially related to the study drug. Data regarding any AE reported prior to day 29 will be gathered until the resolution of the AE. The population that will take part in this study are multi-morbid and suffer from numerous fluctuating chronic symptoms. Recording all symptoms as possible AEs beyond day 29 would likely lead to an inability to identify any meaningful trends in the AE analysis.

Beyond day 29, all SAEs and AESIs (whether serious or non-serious) will continue to be gathered until the final study visit on week 48 or day 337. AESIs have been defined based on potential risks associated with the mAb class of therapeutics administered via intravenous infusion and the data available from the COMET-ICE study, and include the following:

- Hypersensitivity reactions (including skin reactions and bronchospasm) at any time after IMP infusion
- Infusion-related reactions within 24 h after IMP infusion (including pyrexia, chills, dizziness and dyspnoea)

Antibody-dependent enhancement (ADE) of disease in those who reach the primary end-point will be assessed

#### Frequency and plans for auditing trial conduct {23}

No formal audits on behalf of the sponsor are planned, but should a monitoring visit or audit be requested, investigators must make the trial documentation and source data available to the auditor or sponsor’s representative.

#### Plans for communicating important protocol amendments to relevant parties (e.g. trial participants, ethical committees) {25}

Protocol amendments must be reviewed and agreement received from the sponsor for all proposed amendments prior to submission to the REC and/or MHRA. The only circumstance in which an amendment may be initiated prior to REC and/or MHRA approval is where the change is necessary to eliminate apparent, immediate risks to the participants (urgent safety measures). In this case, the accrual of new participants will be halted until the REC and/or MHRA approval has been obtained.

All correspondence with the REC will be retained in the trial master file/investigator site file. A complete list of all protocol amendments will be included in the final clinical study report for each arm of the platform.

#### Dissemination plans {31a}

On completion of each intervention of the trial, the data will be analysed and tabulated, and an intervention-specific final trial report prepared. The results of this trial may be initially disseminated via a press release and then subsequently published and presented at scientific meetings. The results will be summarised in an easily accessible format for patients.

##### Niclosamide and ciclesonide arm

These arms are event-driven, and these arms will close when the required number of symptomatic SARS-CoV-2 infections have been observed. At common close-out, all participants still receiving IMP will be instructed to stop, and a final safety assessment will be conducted 4–6 weeks later. Analyses will be conducted once all study visits are complete.

##### Sotrovimab arm

The analysis of the primary outcome measure will be performed when all randomised patients have completed week 12 or up to 4 weeks post the date of the last confirmed positive SARS-CoV-2 result to allow for symptom collection, for those cases that occur between week 8 and week 12. The efficacy on key secondary outcome measure analyses will be performed when there are a sufficient number of patients that have completed week 16, week 24, week 36 and week 48 (or up to 4 weeks post the date of last confirmed positive SARS-CoV-2 result), using a hierarchical testing procedure. The details will be documented in the statistical analysis plan.

## Discussion

Despite the development of vaccination against SARS-CoV-2 infection transforming the lives of the majority of the population in the pandemic, sub-optimal vaccine responses and vulnerability to SARS-CoV-2 infection remain a major issue for a broad range of immunocompromised individuals. There is an ongoing unmet need for pre-exposure prophylactic strategies for these patients to reduce hospitalisation and death.

This study initially commenced in renal patients only, as in the first wave of infection in April 2020, individuals requiring renal replacement therapy suffered particularly poor outcomes. However, with the vaccine roll out, and sub-optimal vaccine responses becoming apparent across a range of conditions, the patient population eligibility criteria were broadened to include not just renal patients, but also immunology, haematology oncology, rheumatology and organ transplant recipients—any individual with a sub-optimal vaccine response (https://www.ecdc.europa.eu/en/covid-19/high-risk-groups)

The trial has had to be flexible and is designed to be able to adapt to the changing landscape of the COVID-19 pandemic. For example, when initially approved, SARS-CoV-2 vaccination was not even on the horizon and now is the standard of care. As such, the trial protocol has had to adapt to ensure that no patient is denied standard-of-care treatment through participation in the trial. Also, in April 2022, PCR tests were no longer required for confirmation of infection or to access therapeutics for SARS-CoV-2 infection, and therefore, we amended the protocol to permit either PCR or lateral flow test confirmed SARS-CoV-2 infection as the primary endpoint. Every change is carefully considered evaluating the impact on the final data analysis, but in a research environment evolving so rapidly, a reactive approach is necessary to ensure that the trial remains relevant.

The trial design is pragmatic and aims to minimise interactions in the healthcare setting with vulnerable individuals. As such, linkage with routinely collected healthcare data via UKHSA, together with the use of telephone and electronic follow-up, are central to the trial processes. We continue to review emerging literature for potential further agents to add to the platform, both re-purposed drugs and also novel monoclonals and antiviral agents.

## Trial status

Recruitment commenced to the niclosamide arm of the study in February 2021 and that arm is anticipated to close at the end of 2022 when the required number of symptomatic SARS-CoV-2 positive end points have accumulated. As of 31 October 2022, 1611 patients have been randomised (1223 in the UK and 388 in India). Approvals have been in place to add ciclesonide to the platform from May 2021, but it has not been possible to secure a drug supply for the whole trial. The sotrovimab arm opened in August 2022 (after a delay of 8 months to allow for a protocol revision to increase the dose from 500 to 2000 mg in light of emerging variants). As of 31 October 2022, 68 patients have been randomised. Recruitment is expected to complete by Q3 2023. The study investigators are actively scanning emerging data on future potential agents to be trialled in the platform.

## Data Availability

When the database from each arm of the trial is locked, analysed and published, we will endeavour to make data available upon reasonable request and pursuant to contractual agreements.

## Abbreviations

AE: Adverse event
AKI: Acute kidney injury
ALT: Alanine aminotransferase
AR: Adverse reaction
ARDS: Acute respiratory distress syndrome
AST: Aspartate aminotransferase
CCTU: Cambridge Clinical Trials Unit
CI: Confidence interval
COVID-19: Coronavirus-induced disease 2019
DSUR: Development Safety Update Report
ECMO: Extracorporeal membrane oxygenation
eCRF: Electronic case report form
GFR: Glomerular filtration rate
GCP: Good Clinical Practice
ICF: Informed consent form
IDMC: Independent Data Monitoring Committee
IMP: Investigational medicinal product
IRR: Infusion-related reaction
ITT: Intention-to-treat
LFT: Lateral flow test
MHRA: Medicines and Healthcare products Regulatory Agency
NIHR: National Institute of Health Research
ONS: Office National Statistics
PCR: Polymerase chain reaction
PHE: Public Health England
PID: Patient identifiable data
PIS: Patient information sheet
PK: Pharmacokinetic
REC: Research Ethics Committee
SAE: Serious adverse event
SAR: Serious adverse reaction
SARS-CoV-2: Severe acute respiratory syndrome coronavirus 2
SUSAR: Suspected unexpected serious adverse reaction
TMG: Trial Management Group
TNF: Tumour necrosis factor
TSC: Trial Steering Committee
UKHSA: UK Health Security Agency
WHO: World Health Organization
WOCBP: Woman of child-bearing potential
